# CSPG4 CAR-redirected Cytokine Induced Killer lymphocytes (CIK) as effective cellular immunotherapy for HLA class I defective melanoma

**DOI:** 10.1186/s13046-023-02884-x

**Published:** 2023-11-22

**Authors:** Lidia Giraudo, Giulia Cattaneo, Loretta Gammaitoni, Ilenia Iaia, Chiara Donini, Annamaria Massa, Maria Laura Centomo, Marco Basiricò, Elisa Vigna, Alberto Pisacane, Franco Picciotto, Enrico Berrino, Caterina Marchiò, Alessandra Merlini, Luca Paruzzo, Stefano Poletto, Daniela Caravelli, Andrea Michela Biolato, Valentina Bortolot, Elisa Landoni, Marco Ventin, Cristina R. Ferrone, Massimo Aglietta, Gianpietro Dotti, Valeria Leuci, Fabrizio Carnevale-Schianca, Dario Sangiolo

**Affiliations:** 1 Candiolo Cancer Institute, FPO-IRCCS, Strada Provinciale 142 Km 3.95, 10060 Candiolo, TO Italy; 2 Department of Oncology, University of Turin, Regione Gonzole 10, 10043 Orbassano, TO Italy; 3 Division of Surgical Oncology, Department of Surgery, Massachusetts General Hospital, Harvard Medical School, Boston, MA USA; 4 Dermatologic Surgery Section, Department of Surgery, Azienda Ospedaliera Universitaria (AOU) Città Della Salute E Della Scienza, Turin, TO Italy; 5 Department of Medical Sciences, University of Turin, Corso Dogliotti 14, 10126 Turin, TO Italy; 6 Cytoskeleton and Cancer Progression, Department of Oncology, Luxembourg Institute of Health, Luxembourg City, Luxembourg; 7 Faculty of Science, Technology and Medicine, University of Luxembourg, Esch-Sur-Alzette, Luxembourg; 8 Department of Microbiology and Immunology, University of North Carolina, Chapel Hill, NC USA; 9 Department of Surgery, Cedars-Sinai Medical Center, Los Angeles, CA USA

**Keywords:** CIK, CAR, CSPG4, HLA class-I, Immunotherapy, Melanoma

## Abstract

**Background:**

Even acknowledging the game-changing results achieved in the treatment of metastatic melanoma with the use of immune checkpoint inhibitors (ICI), a large proportion of patients (40–60%) still fail to respond or relapse due to the development of resistance. Alterations in the expression of Human Leukocyte Antigen class I (HLA-I) molecules are considered to play a major role in clinical resistance to ICI. Cellular immunotherapy with HLA-independent CAR-redirected lymphocytes is a promising alternative in this challenging setting and dedicated translational models are needed.

**Methods:**

In this study, we propose an HLA-independent therapeutic strategy with Cytokine Induced Killer lymphocytes (CIK) genetically engineered with a Chimeric Antigen Receptor (CAR) targeting the tumor antigen CSPG4 as effector mechanism. We investigated the preclinical antitumor activity of CSPG4-CAR.CIK in vitro and in a xenograft murine model focusing on patient-derived melanoma cell lines (Mel) with defective expression of HLA-I molecules.

**Results:**

We successfully generated CSPG4-CAR.CIK from patients with metastatic melanoma and reported their intense activity in vitro against a panel of CSPG4-expressing patient-derived Mel. The melanoma killing activity was intense, even at very low effector to target ratios, and not influenced by the expression level (high, low, defective) of HLA-I molecules on target cells. Furthermore, CAR.CIK conditioned medium was capable of upregulating the expression of HLA-I molecules on melanoma cells. A comparable immunomodulatory effect was replicated by treatment of Mel cells with exogenous IFN-γ and IFN-α. The antimelanoma activity of CSPG4-CAR.CIK was successfully confirmed i*n vivo,* obtaining a significant tumor growth inhibition of an HLA-defective Mel xenograft in immunodeficient mice.

**Conclusions:**

In this study we reported the intense preclinical activity of CSPG4-CAR.CIK against melanoma, including those with low or defective HLA-I expression. Our findings support CSPG4 as a valuable CAR target in melanoma and provide translational rationale for clinical studies exploring CAR-CIK cellular immunotherapies within the challenging setting of patients not responsive or relapsing to immune checkpoint inhibitors.

## Background

Over the last ten years the emergence of immunotherapy with immune checkpoint inhibitors (ICI) and targeted therapies (BRAF and MEK inhibitors) has revolutionized the management of metastatic melanoma, leading to a remarkable improvement in the prognosis and overall survival of patients [1–3]. Even acknowledging these important successes, approximatively 40–60% of patients still do not derive long-lasting benefit from therapy with ICI, by either failing to respond from the beginning of the treatment or by developing an acquired resistance, which ultimately leads to tumor relapse [4].

Among the multiple and heterogeneous mechanisms of resistance to ICI, alterations in the expression of Human Leukocyte Antigen class I (HLA-I) molecules and Antigen Processing Machinery (APM) components are considered one of the major drivers, as they cause disruption of antigen presentation by cancer cells and escape from T cell immune recognition and elimination [5, 6]. Indeed, genetic aberrations of HLA-I and β2 microglobulin (β2m) have been associated with disease progression and poorer overall survival in melanoma patients treated with ICI [7, 8].

Cellular immunotherapies, and in particular HLA-independent approaches, represent promising alternative therapeutic options to be explored for patients with metastatic melanoma not responding to ICI. Within this scenario, strategies exploiting lymphocytes engineered with a Chimeric Antigen Receptor (CAR) hold great promises, and many preclinical research efforts and clinical studies are currently ongoing to extend to solid tumors the positive results achieved in the field of hematological malignancies [9–13].

Besides conventional T lymphocytes as platform to design CAR-based immunotherapies, there is growing interest in exploring alternative immune effectors intrinsically endowed with antitumor activity, which may present functional advantages when challenged against solid tumors [14–18]. To this end, Cytokine Induced Killer lymphocytes (CIK) represent an extremely valuable and intriguing option.

CIK are ex vivo expanded T lymphocytes, characterized by a T and natural killer (NK) cell-like phenotype and endowed with an intense HLA-independent antitumor activity, which has been largely demonstrated in preclinical studies with different types of hematologic and solid malignancies, including melanoma [19–23]. The intrinsic HLA-independent cytotoxic ability of CIK is mainly mediated by the NKG2D receptor, which binds stress-inducible molecules (MIC A/B, ULBPs) selectively expressed by cancer cells [24]. The cell subset of CIK co-expressing CD3 and CD56 molecules is present at variable rates and is considered the main driver of CIK-mediated antitumor activity [25]. Initial promising results observed in clinical trials support the excellent safety profile of CIK and provide evidence of clinical activity in several settings of hematological and solid malignancies [26, 27].

CIK may represent an ideal platform for CAR-based immunotherapies, as they would exert a dual potent HLA-independent antitumor activity, conjugating their intrinsic NKG2D-mediated cytotoxicity with the CAR-specific recognition and elimination of cancer cells. First evidence of feasibility, safety, and efficacy of CAR CIK-based immunotherapy derives from clinical trials including patients with lymphoid and myeloid leukemia [28–30].

We previously reported proof of concept that CAR.CIK can be effectively generated from patients’ circulating precursors, displaying intense preclinical activity against multiple histotypes of soft tissue sarcomas, superior to the one observed with conventional unmodified CIK [31, 32].

The successful application of a CAR-based immunotherapy highly depends on the selection of a suitable target tumor antigen (TA), which ideally should be exclusively expressed on cancer cells with limited heterogeneity and play relevant biological roles in tumors. We selected the surface protein chondroitin sulfate proteoglycan 4 (CSPG4) as target TA of our CAR.CIK-based strategy, since it has been reported to be functionally involved in processes of tumor growth, progression and metastatic spread, it is over expressed on cancer cells with limited distribution in normal tissues, and it has been detected on both differentiated cancer cells and cancer initiating cells in several types of solid tumors [33–36]. In our previous studies, we demonstrated that CAR.CIK redirected against CSPG4 mediated intense antitumor activity in vitro and in vivo against soft tissue sarcomas [31].

In this study, we investigated the preclinical activity of patient-derived CSPG4-CAR.CIK against patient-derived melanoma cells, focusing on models with defective expression of HLA-I molecules, as this setting might provide a representative perspective of the challenging clinical scenario.

## Methods

### Aim and design of the study

The aim of this study was to investigate the efficacy of CSPG4-CAR.CIK-based immunotherapy in a preclinical setting of metastatic melanoma, with specific focus on tumors featuring low expression or complete loss of HLA class I molecules. In our study we successfully generated CSPG4-CAR.CIK from patients with metastatic melanoma and tested their antitumor activity ***i)*** against patient-derived melanoma cell lines in vitro and ***ii)*** in a xenograft murine model generated by grafting an HLA-defective patient derived melanoma cell line in NSG mice.

### Melanoma cell lines

Melanoma cell lines were generated from patients with advanced stage IV melanoma, when patients where off or before starting of systemic treatments. Melanoma tissue samples surgically removed from 24 patients were collected at the Candiolo Cancer Institute-IRCCS (Candiolo, Turin, Italy). Patient-derived samples were collected according to an internal institutional review board (IRB)-approved protocol (protocol no. 196/2010, no. 225/2015). Tumor tissue samples were processed by mechanic and enzymatic dissociation to obtain single cell suspensions and characterized for the expression of the main melanoma surface antigens, as previously described [37]. Melanoma (Mel) cell lines were cultured at 37 °C and 5% CO_2_ utilizing KO DMEM F12 (Gibco, Thermo Fisher Scientific) or RPMI-1640 (Sigma-Aldrich) culture medium, supplemented with 10% FBS (Sigma-Aldrich) and Penicillin (100U/ml)-Streptomycin (100 µg/ml) (Sigma-Aldrich). Sanger sequencing was performed as previously described for β2m [38] and for HLA-I [39] genes on the DNA purified from M017. The A375 melanoma cell line was obtained from the American Type Culture Collection (ATCC).

### Generation of patient derived CSPG4-CAR.CIK

CIK were generated from patient-derived Peripheral Blood Mononuclear cells (PBMCs) collected according to an internal institutional review board (IRB)-approved protocol (protocol no., 225/2015). PBMCs were isolated by density gradient centrifugation (Lymphoprep™, Aurogene) and seeded in 25cm^2^ flasks at the concentration of 2 × 10^6^ cells/ml in RPMI-1640 culture medium supplemented with 10% FBS (Sigma-Aldrich), Penicillin (100U/ml)-Streptomycin (100 µg/ml), and in the presence of IFN-γ (1000 U/ml, Miltenyi Biotec). On day 1 Anti-Biotin MACSBead particles loaded with anti-CD2, anti-CD3, and anti-CD28 antibodies (Miltenyi Biotec), and human interleukin 2 (IL-2) (300 U/ml, Miltenyi Biotec) were added to the culture medium to induce PBMCs activation. On day 2 PBMCs were seeded in a non-treated 24-well tissue culture plate previously coated with RetroNectin (25 µg/well, Takara Shuzo, Otsu, Japan), and transduced with 0.5 ml of retroviral supernatant. The viral vector, kindly provided by Dr. G. Dotti (University of North Carolina, USA), encodes a CAR generated from the CSPG4-specific scFv 763.74 and comprised of the 4-1BB costimulatory domain. CSPG4-CAR CIK were expanded in vitro according to a standard protocol, as previously described [25]. CIK engineered with a CD19-specific CAR, generated from the CD19-specific scFv MFC63, were generated following the same procedure.

### Flow cytometry

To assess NKG2D ligands expression, melanoma cells were detached utilizing Accutase Cell dissociation reagent (Thermo Fisher Scientific), washed with PBS, and incubated for 30-min at 4 °C with the mouse anti-human monoclonal antibodies (mAbs) specific for MIC A/B (PE-conjugated anti-MIC A/B, BD Pharmingen™, dilution 1:100) and ULBP-2–5-6 (APC-conjugated anti-ULBPs, R&D Systems®, dilution 1:100). Melanoma cells were then washed with PBS and analyzed by flow cytometry. Mouse anti-human primary mAbs specific for CSPG4 (mix of 763.74, 225.28, D2.8.5-C4B8), HLA-ABC (TP 25.99.8.4), β2microglobulin (NAMB-1), LMP2 (SY-1), LMP7 (HB2), LMP10 (TO-7), TAP1 (NOB1), TAP2 (NOB2), Erp57 (TO-2), and Tapasin (TO-3) were kindly provided by Dr. S. Ferrone (Massachusetts General Hospital, Boston, USA). Melanoma cells were detached utilizing Accutase Cell dissociation reagent (Thermo Fisher Scientific), washed with Stain Buffer (BD Pharmingen™), and incubated with primary mAbs (10ug/ml) for 45 min at 4 °C. To detect the expression of intracytoplasmic molecules, melanoma cells were fixed with BD Cytofix™ Fixation Buffer for 20 min at 4 °C, washed twice with BD Stain Buffer, permeabilized with BD Phosflow™ Perm Buffer III (BD Pharmingen™), and incubated with primary mAbs. Following incubation with the primary antibody, cells were washed and incubated with a PE-conjugated Goat anti-Mouse secondary antibody (BD Pharmigen). A Quantum™ R-PE Molecules of Equivalent Soluble Fluorochrome (MESF) kit (Bangs Laboratories) was utilized to quantify the expression level of HLA class I molecules on melanoma cells.

CIK immunophenotype was assessed in the third week of expansion. The following mAbs were utilized: anti-human CD3 FITC (1:100, BD Pharmingen), anti-human CD8 PE (1:100, MACS Miltenyi Biotec), anti-human CD56 PE (1:100, MACS Miltenyi Biotec), anti-human NKG2D APC (1:100, MACS Miltenyi Biotec), anti-human CD45RA FITC (1:100, Miltenyi Biotec), anti-human CD45RO PECy5 (1:100, Miltenyi Biotec), anti-human CD62L PE (1:100, Miltenyi Biotec), anti-human CXCR3 PE (1:100, BD Pharmingen™). The percentage of CAR expression was detected with an APC-conjugated mAb specific for the IgG1/ CH2CH3 spacer (The Jackson Laboratory).

Samples were analyzed utilizing a CyAn ADP Flow Cytometer (Beckman Coulter Inc., USA) and analyzed with Summit v4.3 Software (DAKO, CA, USA).

### Treatment of melanoma cell lines with Interferons (IFNs)

Melanoma cells were seeded in 100 mm cell culture dishes and treated with IFN-α (1 × 10^4^ IU/ml, Human IFN-α2b, research grade Milteny Biotech), or IFN-γ (1 × 10^3^ IU/ml, Human IFN-γ1b, premium grade, Miltenyi Biotec). Untreated cells were cultured in parallel as a control. Following a 48-h incubation, melanoma cells were harvested and stained with mAbs specific for HLA class I molecules and APM components, as previously described.

### In vitro cytotoxicity assays

The antitumor activity of CSPG4-CAR.CIK was assessed in vitro against Mel cell lines by flow cytometry. Target cells were labeled with the CFSE (5,6-carboxyfluorescin diacetate succinimidyl ester) fluorescent dye (Molecular Probes) according to the manufacturer’s protocol. Melanoma cells were then seeded in 48 well tissue culture plate at the concentration of 20.000 cells/well, and cocultured with either CSPG4-CAR.CIK, CD19-CAR.CIK or unmodified CIK at different effector (CIK) to target (tumor) (E/T) ratios (10/1, 5/1, 2/1, 1/1, 1/2, 1/4, 1/8). Melanoma cells alone were cultured in parallel as a control of the spontaneous target cell death. Following a 48-h incubation, the percentage of viable melanoma cells was assessed by flow cytometry and determined by gating on the CFSE positive/DAPI negative cell population. Specifically, CIK and melanoma cells were collected, washed with PBS, and incubated with DAPI (BD Biosciences, 1 µg/ml, 1 min incubation on ice). The rate of CIK-mediated killing was calculated according to the formula: (experimental mortality – spontaneous mortality) / (100 – spontaneous mortality) × 100.

### In vivo experiments

In vivo experiments received approval by the competent committee and internal review board (auth. no. 178/2015-PR).

#### Xenograft murine model

Twenty-four female NOD-*scid* IL2Rg^null^ mice (Charles River) were subcutaneously grafted with the patient-derived Mel cell line M017 (850.000 cells/mouse resuspended in PBS and BD Matrigel Basement Membrane Matrix (Becton Dickinson) at 1:1 ratio). When tumors became palpable, mice were randomly assigned to 3 treatment groups (8 mice/group). Mice received either CSPG4-CAR.CIK, unmodified CIK or were left untreated as a control of the spontaneous growth of the tumor. CIK (3 × 10^6^ cells/mouse) were administered by tail vein injection every 3 days for a total of 5 infusions. Tumor growth was monitored twice weekly by caliper measurement, and tumor volume was determined according to the formula V = 4/3 × π x(*a*/2)^2^ x (*b*/2)^2^ where *a* and *b* represent the length and the width diameter of the tumor, respectively. When tumors of mice included in the untreated control group reached 1.5 cm in diameter, all mice were sacrificed, and tumors harvested for further analysis.

#### Ex vivo imaging by detection of fluorescent glucose

Twenty-four hours before the end of the experiments, mice were intravenously injected with the fluorescent probe XenoLight RediJect 2-DeoxyGlucosone (DG)-750 (5 nmol/mouse) (PerkinElmer). Following mice sacrifice, tumors were collected, and the uptake of fluorescent glucose was determined by Living Image Software and IVIS Spectrum CT (Caliper Corporation, PerkinElmer Company).

### Statistical analysis

Data were analyzed by using GraphPad Prism 9 software (VERSION 9.5.1) and presented as mean values ± SD of at least two independent experiments. One-way ANOVA with Tukey’s multiple comparison test and two-way ANOVA with Sidak’s multiple comparison test were utilized to determine statistical significance in the comparison among groups. Two-way repeated measures ANOVA with Sidak’s multiple comparison test was utilized to compare tumor volumes. Significance is represented in graphs as *ns* for not significant, ** for *p* ≤ *0.01*, *** for *p* ≤ *0.001*, **** for *p* ≤ *0.0001*.

All data generated in this study are available from the authors upon reasonable request.

## Results

### Heterogeneous expression of HLA class I molecules on patient-derived melanoma cell lines

**Fig. 1:**
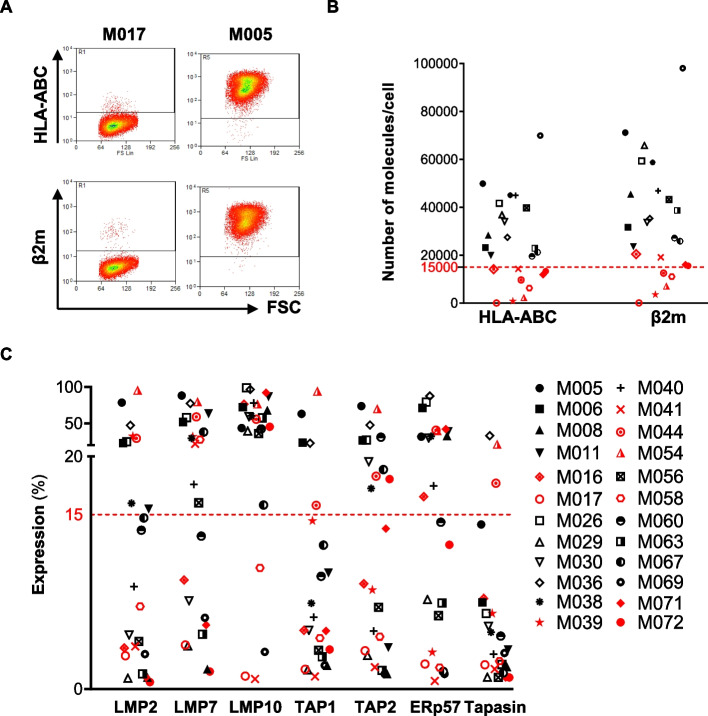
Heterogeneous expression of HLA-I molecules and Antigen Processing Machinery (APM) components on melanoma cell lines. Patient-derived melanoma cell lines (Mel) (*n* = *24*) were characterized by flow cytometry for the expression of HLA class I molecules (HLA-ABC and β2 microglobulin (β2m)) and APM components (LMP2, LMP7, LMP10, TAP1, TAP, Erp57, Tapasin). **A** Representative dot plots showing the lack of expression of both HLA-ABC and β2m molecules on the Mel M017; the HLA-I positive M005 Mel cell line is shown as control. **B** Quantification of the number of HLA-I molecules expressed on the surface of melanoma cells. Red symbols indicate Mel with a particularly low expression level (less than 15000 molecules/cell) of HLA-ABC (9/24 Mel) and β2m (5/24 Mel). HLA-ABC and β2 expression level was equal to or higher than 15000 molecules in 15/24 melanoma (black symbols). **C** Heterogeneous expression of the immunoproteasome subunits (LMP2, LMP7, LMP10) and of the protein loading complex components (TAP1, TAP2, Erp57, Tapasin) on Mel. Most of the analyzed cell lines showed a low expression level (defined as less than 15% of positive cells) of LMP2 (15/24), TAP1 (18/23), and Tapasin (21/24). TAP2, LMP7, LMP10 and Erp57 expression was more conserved, as an expression level higher than 15% was detected in 11/24, 14/24, 20/24, and 13/24 Mel respectively

As a first step, we characterized for the expression of the HLA class I (HLA-I) complex 24 patient-derived melanoma cell lines (Mel) generated from metastatic tissues surgically removed from patients with advanced stage melanoma. A positive membrane expression of the HLA-I complex, which is composed of HLA-ABC and β2 microglobulin (β2m) molecules, was detected in 23/24 Mel, whilst one Mel (M017) resulted negative. By sanger sequencing analysis of M017 cells, we identified a homozygous single nucleotide variant (SNV) mutation in the first codon of the β2m gene. Such mutation, identified as p.Met1Ile, leads to the loss of the first Methionine, resulting in the absence of the β2m protein, which ultimately results in the lack of translocation of the HLA-I complex on the cell membrane (Fig. 1A).

To better discriminate between different HLA-I expression levels within Mel, we quantified the number of HLA-ABC/β2m molecules present on the surface of melanoma cells. The density of HLA-I molecules per cell resulted heterogeneous. Based on this heterogeneous distribution, we established a threshold of 15,000 molecules per cell, to define a first group of Mel displaying a high HLA-ABC molecule density (15/24), and a second one characterized by a particular low expression of HLA-ABC (9/24) (Fig. 1B).

In the same 24 Mel, we reported a heterogeneous baseline expression of some key components of the intracellular Antigen Processing Machinery (APM), including the subunits of the immunoproteasome (LMP2, LMP7, LMP10) and the components of the protein loading complex (TAP1, TAP2, Erp57, Tapasin), responsible of the immunogenic peptide processing, loading on the HLA-I complex and exposure on the cell membrane. In general, we observed a low basal expression (defined as less than 15% of positive cells) of these proteins (Fig. 1C).

### Generation and phenotypic characterization of patient-derived CSPG4-CAR.CIK

**Fig. 2:**
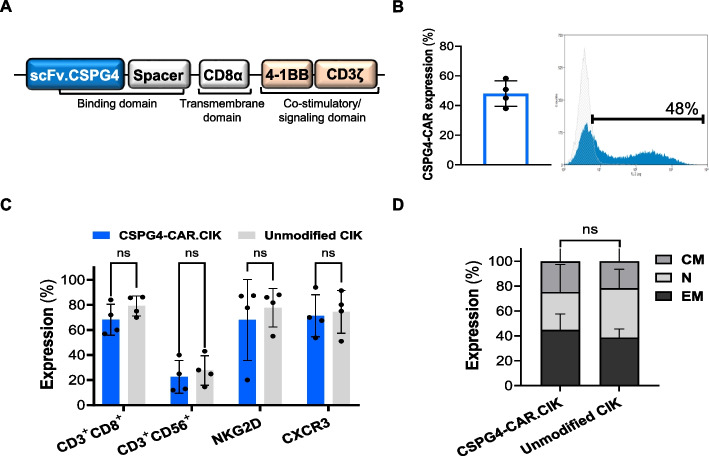
Characterization of CSPG4-CAR.CIK generated from patients with metastatic melanoma. PBMCs derived from melanoma patients (*n* = *4*) were engineered with a CSPG4-specific CAR. Following ex vivo expansion, CSPG4-CAR.CIK were characterized by flow cytometry for transduction efficiency and expression of the main CIK markers. **A** Schematic representation of the CAR construct, composed by i) an extracellular domain derived from the single chain variable fragment (scFv) of the CSPG4-specific mAb 763.74, ii) a transmembrane domain, and iii) an intracellular signaling region which includes the 4-1BB co-stimulatory domain and CD3ζ signaling domain. **B** Mean transduction efficiency of CSPG4-CAR.CIK lymphocytes and representative flow cytometry histogram. **C** Phenotypic characterization of CSPG4-CAR.CIK compared to unmodified CIK. **D** Characterization of CSPG4-CAR.CIK for the expression of effector memory (EM: 44 ± 13%), naïve (N: 30 ± 22%) and central memory (CM: 24 ± 14%) markers. Statistical analysis was performed by two-way ANOVA multiple comparison test. Results are shown as mean ± SD

CSPG4-CAR.CIK were generated from peripheral blood mononuclear cells (PBMCs) obtained from metastatic melanoma patients (*n* = *4*), and ex vivo expanded according to a previously established protocol [25]. The CAR construct we utilized is specific for the CSPG4 target TA and comprised of the 4-1BB costimulatory domain. The mean transduction efficiency of the generated CSPG4-CAR.CIK was 48 ± 8% (Fig. 2A, B). Mature CSPG4-CAR.CIK mainly displayed a CD3^+^ CD8^+^ phenotype (71 ± 13%), with a mixed T-NK phenotype, as shown by the presence of a cell subpopulation expressing the CD56 molecule (CD3^+^ CD56^+^, 22 ± 13%). Notably, CSPG4-CAR.CIK displayed a high membrane expression of NKG2D and CXCR3 receptors (68 ± 32% and 71 ± 2%, respectively) (Fig. 2C).

Among the bulk population of CAR.CIK, the effector memory (EM) subset was the most represented (CD45RA^−^ CD45RO^+^ CD62L^−^, 44 ± 13%), followed by naïve (N) (CD45RA^+^ CD45RO^−^ CD62L^+^, 30 ± 22%) and central memory (CM) (CD45RA^−^ CD45RO^+^ CD62L^+^, 24 ± 14%) T cells.

We confirmed that the genetic manipulation of CIK did not affect their baseline phenotypic features, as CSPG4-CAR.CIK were comparable with unmodified CIK, utilized as control (Fig. 2C, D).

### CSPG4-CAR.CIK can effectively kill in vitro patient-derived melanoma cells, independently from the HLA class I expression level

**Fig. 3:**
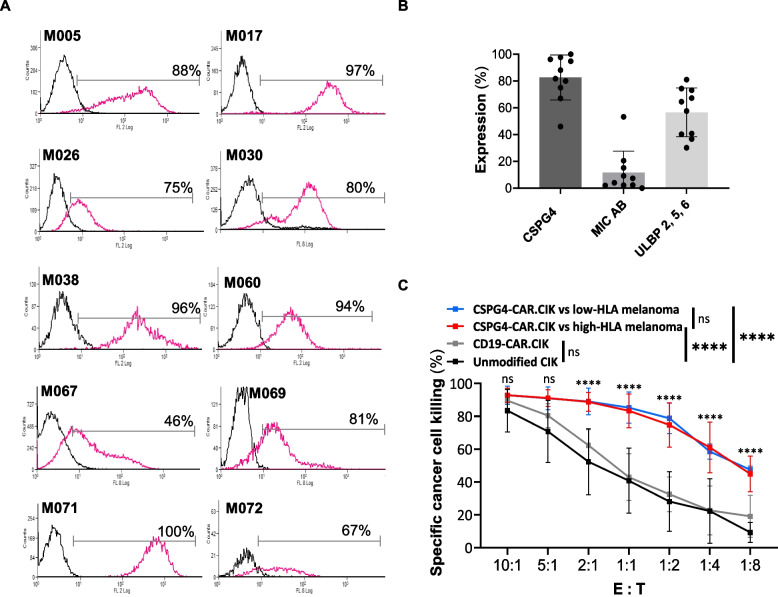
In vitro antimelanoma activity by CSPG4-CAR.CIK against melanoma cells, independently from HLA class I expression. Patient-derived Mel (*n* = *10*) were characterized by flow cytometry for CSPG4 expression and for the presence of MICA/B and ULBP2-5–6 ligands. The same Mel were then utilized as targets to test in vitro the antitumor activity of CSPG4-CAR.CIK. **A** Flow cytometry histograms showing CSPG4 expression on Mel. **B** Mean expression level of CSPG4 (82 ± 17%), MIC A/B (11 ± 16%), and ULBP2-5–6 (56 ± 18%) on melanoma cells, with each symbol representing a single Mel. **C** Specific tumor lysis of Mel with high (*n* = *7*) or low/absent (*n* = *3*) HLA-I expression, co-cultured with CSPG4-CAR.CIK, CD19-CAR.CIK or unmodified CIK at different E/T ratios. Percentage of tumor cell killing was determined by flow cytometry following a 48-h incubation period. The extent of CSPG4-CAR.CIK-mediated killing was significantly higher compared to unmodified (*p* < *0.001*) and CD19-CAR CIK (*p* < *0.0001*), utilized as controls. No significant difference was observed when comparing unmodified CIK with CD19-CAR.CIK (*p* = 0.123), or the activity of CSPG4-CAR.CIK vs either high- or low-HLA melanoma (*p* = 0.995) at all E:T ratio. Statistical significance showed for each E:T ratio refers to the comparison between the killing mediated by CSPG4-CAR.CIK and the killing mediated by unmodified CIK. Comparisons between other groups are indicated in the figure legend. Statistical analysis was performed by two-way ANOVA multiple comparison test. Results are shown as mean ± SD

To support the rationale of a CSPG4-redirected CAR-based immunotherapy, we assessed CSPG4 frequency and level of expression on 10 Mel from the previously characterized cohort of patient-derived cell lines. As demonstrated in Fig. 3A, we confirmed that CSPG4 was highly and homogeneously expressed on all the analyzed Mel with a mean of 82 ± 17%. In addition, the same Mel cell lines expressed at least one of the main ligands of the NKG2D receptor (MIC A/B and ULBP2-5–6), with a higher rate of Mel expressing ULBP2-5–6 compared to MIC A/B (Fig. 3B). The presence of these molecules on tumor cells is crucial for CIK to exert their intrinsic HLA-independent cytotoxic activity, which is mainly mediated by the NKG2D receptor.

To test in vitro the antitumor potential of CSPG4-CAR.CIK, we selected the CSPG4-expressing Mel described in Fig. 3A, as they present variable HLA-I expression levels (high expression: M005, M026, M038, M030, M067, M069, low or negative expression:M071, M072, M017). CSPG4-CAR.CIK effectively eliminated melanoma cells, displaying a significantly higher cytotoxic activity compared to unmodified CIK and control CD19-CAR.CIK, especially at low E/T ratios (percentage of tumor specific lysis at E/T = 1/4 for CSPG-CAR.CIK: 66 ± 15% vs unmodified CIK: 22 ± 20%; percentage of tumor specific lysis at E/T = 1/8 for CSPG-CAR.CIK: 46 ± 7% vs unmodified CIK: 9 ± 6%). Importantly, we demonstrated that all the tested Mel were equally killed by CSPG4-CAR.CIK, independently from their HLA-I expression profile (Fig. 3C).

### Treatment with IFN-α and IFN-γ enhances the expression of HLA class I molecules and APM components on melanoma cells

**Fig. 4:**
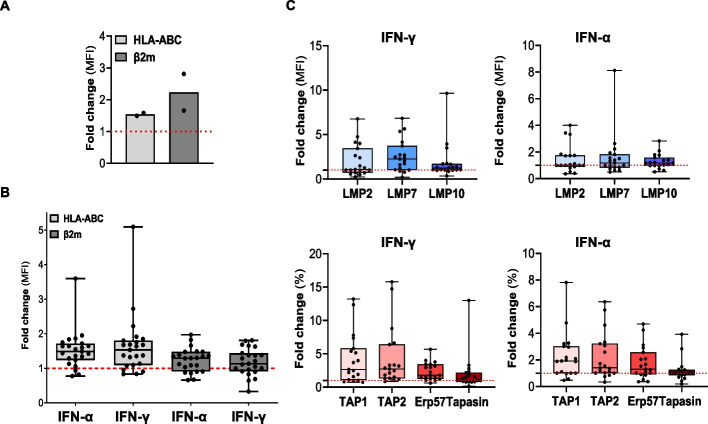
Immunomodulatory effects induced by interferons on HLA class I and APM expression on melanoma cells. The expression level of the HLA-I complex and APM components was assessed by flow cytometry on melanoma cells after exposure to CSPG4-CAR.CIK-conditioned medium or following treatment with IFN-α (1 × 10^4^ IU/ml) and IFN-γ (1 × 10^3^ IU/ml). **A** Expression of HLA-ABC and β2m molecules on two melanoma cell lines (M005 and A375) cultured for 48 h in the presence of CSPG4-CAR.CIK-conditioned medium. **B** Assessment of HLA-ABC and β2m molecules expression level on Mel (*n* = *23*) following treatment with IFNs. Results are shown as mean fluorescence intensity (MFI) fold change, quantified utilizing Molecules of Equivalent Soluble Fluorochrome (MESF). Any fold change > 1 was considered as an increase of the expression of the molecule of interest. **C** Expression of immunoproteasome (LMP2, LMP7, LMP10) and protein loading complex (TAP1, TAP2, Erp57, Tapasin) molecules on Mel (*n* = *19*) following treatment with IFNs. Results are shown as mean fold change of the percentage of positive cells. Any fold change > 1 was considered as an increase of the expression of the molecule of interest. Untreated melanoma cells were utilized as control of the basal expression level

We investigated whether, in addition to the direct tumor killing activity, CSPG4-CAR.CIK could also modulate and favor HLA-I expression on melanoma cells. To this end, Mel M005 and the A375 melanoma cell lines were cultured in the presence of CSPG4-CAR.CIK-conditioned medium for 48 h. At the end of the incubation, we observed that expression levels of HLA-ABC and β2m were higher on melanoma cells cultured in the presence of the conditioned medium, compared to the control (1.54- and 2.24-fold change in mean fluorescence intensity (MFI), respectively) (Fig. 4A).

Within the cytokines secreted by CAR.CIK, we hypothesized that this effect could mainly be due to the high release of interferons (IFNs), specifically IFN-γ and IFN-α, as it was previously reported by our group [31]. To confirm our hypothesis, we treated in vitro 23 Mel with IFN-α and IFN-γ to assess their immunomodulatory effects.

Exposure of melanoma cells to IFNs markedly enhanced the basal expression of HLA-ABC and β2m compared to untreated cells utilized as control of the basal expression level (mean fold change respectively of 1.53 ± 0.56 and 1.24 ± 0.34 with IFN-α; 1.65 ± 0.88 and 1.18 ± 0.39 with IFN-γ) (Fig. 4B).

Following the same experimental design, we investigated whether the immunomodulatory activity mediated by IFN-α and IFN-γ also had an impact on the expression level of APM components on melanoma cells. We observed a general trend of upregulation induced by both IFNs in the expression of the immunoproteasome and the protein loading complex proteins in the treated Mel (Fig. 4C).

Our findings report the immunomodulatory effects induced by interferons on each analyzed molecule. Overall, we demonstrated a general trend of upregulation in the expression of HLA class I molecule and APM components on melanoma cells.

### CSPG4-CAR.CIK effectively controlled tumor growth of HLA-defective melanoma in vivo

To confirm in vivo the strong antimelanoma activity mediated by CSPG4-CAR.CIK in vitro, we tested their antitumor potential in a xenograft murine model of melanoma, generated by subcutaneous grafting of M017 cells in NSG mice. We specifically selected this Mel since it is HLA-defective and therefore representative of a challenging clinical setting.

**Fig. 5:**
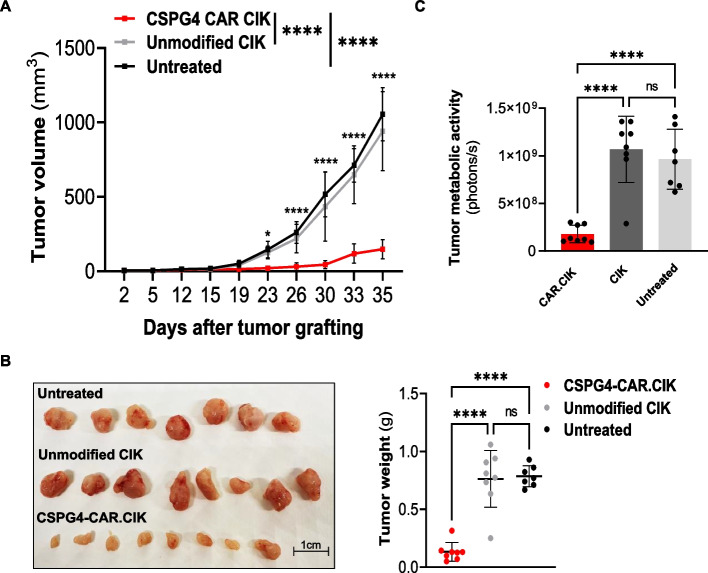
Effective tumor inhibition by CSPG4-CAR.CIK in a xenograft murine model of HLA-defective melanoma. The M017 HLA-defective Mel was selected to generate a xenograft murine model of melanoma and to test in vivo the cytotoxic activity of CSPG4-CAR.CIK. M017 cells (850.000 cells/mouse) were subcutaneously grafted in NSG mice (*n* = *24*). One group of mice was treated with CSPG4-CAR.CIK, a second group received unmodified CIK and a third group was left untreated as control of the spontaneous tumor growth. CIK and CSPG4-CAR.CIK (3 × 10^6^ cells/mouse) were administered every two days, for a total of five times. Tumor growth was monitored by caliper measurement. The endpoint of the experiment was tumor eradication and mice were sacrificed 35 days after grafting melanoma cells. **A** Effective control of tumor growth mediated by CSPG4-CAR.CIK up to 2 weeks after the last administration, significantly higher compared to unmodified CIK and untreated control (*p* < *0.0001*). Statistical significance showed at each timepoint refers to the comparison between tumor volume of mice treated with CSPG4-CAR.CIK and mice treated with unmodified CIK. **B** Representative pictures and weight measurement of tumors collected from the different treatment groups. **C** *Ex viv*o measurement of fluorescent glucose uptake by tumor cells, utilized as indicator of the tumor metabolic activity. Statistical analysis was performed by two-way ANOVA multiple comparison test for Fig. A, by one-way ANOVA multiple comparison test for Fig. B and C. Data are shown as mean ± SD

Two days after tumor cell grafting, mice were treated with CSPG4-CAR.CIK every three days for a total of five times (3 × 10^6^ cells/mouse each administration). A group of mice treated with unmodified CIK was included as control. As shown in Fig. 5A, CSPG4-CAR.CIK markedly delayed tumor growth to a significantly higher extent compared to unmodified CIK and untreated mice (*p* < *0.0001*). This evidence was confirmed by the measurement of tumor weight, which was significantly lower in mice treated with CSPG-CAR.CIK (*p* < *0.0001*) (Fig. 5B). In addition, we quantified ex vivo the metabolic activity of freshly collected tumors by measuring the glucose uptake of melanoma cells. Melanoma treated with CSPG4-CAR.CIK showed a significantly lower metabolic rate compared to tumors treated with unmodified CIK (1.8 × 10^8^ ± 9.2 × 10^7^ and 1.1 × 10^9^ ± 3.5 × 10^8^ photons/sec, respectively, *p* < *0.0001*) (Fig. 5C).

## Discussion

In this study we reported the intense preclinical activity of anti-CSPG4 redirected CAR.CIK lymphocytes against melanoma cells, including those with defective expression of HLA-I molecules.

Melanoma relapsing or not responding to immunotherapy with ICI, and not eligible to molecular targeted treatments, have a very dismal prognosis and are in urgent need of innovative therapeutic approaches. The defective expression of HLA-I molecules by tumor cells was a central issue in the rationale framing of our study, as it is considered one of the main causes associated with resistance to ICI. For this reason, cellular immunotherapies not relying on HLA presentation of tumor antigens (TAs) represent a promising strategy to explore and promote in this challenging clinical setting.

The main findings and messages emerging from our study confirm that CSPG4 can be considered a suitable CAR-target TA in melanoma. Furthermore, they support the application of CIK as a valuable platform for CAR engineering and demonstrate the effectiveness of such approach in settings of HLA deficiency with the connected relevance in clinical perspective.

The intense expression of CSPG4 by all our patient-derived melanoma cell lines was observed regardless of the HLA deficiency degree and allowed the strong CAR.CIK-mediated killing, even in challenging conditions of high tumor cell density (low effector to target ratios). This finding is particularly relevant from a clinical perspective, as it will be conceivable that a CAR-based therapy may be initially tested in patients with advanced stage disease, likely reproducing an unfavorable ratio between the incoming CAR.CIK and host tumor cells. The clinical translation of CSPG4-CAR.CIK will require the design of a Phase I trial to demonstrate the safety of this approach, although encouraging premises come from previous studies, including ours, showing that the membrane expression of CSPG4 on normal tissues is lower compared to the one observed in tumors. Preclinical data support the possibility to exploit a therapeutic frame based on the higher expression levels of CSPG4 on tumor cells to segregate the antitumor effects by potential off-tumor toxicities. The biological relevance of CSPG4 in several crucial tumor processes, like tumorigenesis/stemness, aggressiveness and metastatic spread has been reported in melanoma and other cancer settings [33–37, 40], further valuing the potential interest of its targeting for clinical applications.

The majority of current applications with CAR engineered lymphocytes rely on the use of T cells as immune effectors [10]. The proposal of CIK is to be intended as an integrative tool, exploiting the additional intrinsic NKG2D-mediated killing mechanism, which would allow to counteract immune evasion when CAR-targeted antigens are not homogeneously expressed on tumors. We demonstrated that the genetic manipulation of CIK did not affect their phenotype, which was fully comparable with the unmodified controls. The reduced lifespan of CAR.CIK, as compared with CAR T, may have positive implications in terms of safety in cases of unexpected toxicities, also addressing some concerns about the risk of insertional mutagenesis consequent to the engineering with a viral vector. Furthermore, the high expansion rates and cost/effective ex vivo production of CIK enhances the availability of this cell product, allowing the design of clinical protocols encompassing multiple infusions of CAR.CIK.

Within our study we focused on the impact of melanoma HLA deficiencies on the proposed CAR.CIK-based immunotherapy. A quantitative analysis showed a heterogeneous distribution in the expression of HLA-I molecules and APM components in patient-derived melanoma cell lines, including a subset of melanoma with very low levels of membrane HLA-I expression, that would not have been detected by a qualitative assessment only. Of course, there is not a recognized or validated threshold to clearly define high and low HLA-I levels. Therefore, we empirically grouped our melanoma samples based on their segregation and clusterization when analyzed for the number of HLA-I expressed molecules. There is currently increasing interest in the evaluation of HLA expression on tumor cells, given its importance in the context of treatment with ICI. Our data support such warranting and underline the importance of a quantitative evaluation of protein expression at the membrane level.

The antimelanoma activity of CSPG4-CAR.CIK was intense regardless of HLA-I expression level on melanoma cells, including a patient-derived melanoma cell line lacking HLA-I surface expression due to a mutation in the β2m gene. Patients with similar deficiencies have been reported as resistant to immunotherapy with ICI and this could be the prospective clinical setting where to consider the exploration of HLA independent CAR-based cellular immunotherapies [6]. The efficacy of CAR.CIK was confirmed in vivo with a xenograft murine model generated with the same HLA-deficient melanoma. Such model reflects a particularly challenging and even more relevant setting, as this melanoma, besides its HLA-deficiency, was almost insensible to the HLA-independent in vitro and in vivo antitumor activity mediated by unmodified CIK. We do not know the mechanistic reasons underlying such intrinsic resistance, but it is not due to the lack of NKG2D ligands expression. It is our hypothesis that the intrinsic antitumor activity of CIK might be damped by the expression of immunosuppressive pathways on tumor cells, and further dedicated investigations are currently going in this direction. In addition, even acknowledging the limitations of the murine model utilized in our study, we did not observe any sign of toxicity associated with CAR.CIK administration.

A possible speculation arising from our findings is related to the potential beneficial bystander effects of CAR.CIK, derived from their intense release of interferons (IFNs). Indeed, the expression and integrity of HLA-I molecules and APM components are tightly connected and regulated by the interferon pathway. Previous works, including studies from our group, reported the intense IFN-γ production by CIK and CAR.CIK [31, 41]. Here we confirmed that treatment with IFNs enhanced the expression of HLA-I and APM molecules on melanoma cells. CIK-based immunotherapy, besides the direct tumoricidal activity, may prompt indirect positive effects by enhancing the tumor antigen presentation capability and consequently supporting or restoring the adaptive antitumor immune response.

The evidence that the production of IFN-γ by CAR.CIK is stimulated by the interaction with the target TA supports the hypothesis that IFN-γ release occurring in patients after CAR.CIK infusion would be preferentially located at tumor sites, further limiting the risks of IFN-γ-associated systemic effects [41].

The concentrations of IFNs used in our experiments have been empirically chosen, based on previous literature and to preserve the modulatory effect without inducing excessive tumor cell toxicity [41]. Such doses were intended to generate a proof of principle and are likely to be higher as compared with the reported spontaneous CIK production which is, still capable of inducing immunomodulatory effects, as observed when melanoma cells were cocultured with CAR.CIK conditioned medium.

Overall, our findings strongly support the rationale to utilize CIK as a valuable platform for CAR engineering and provide reliable translational bases to explore anti-CSPG4 CAR-based immunotherapies in clinical studies within the challenging setting of patients harboring HLA-defective melanoma who do not benefit from treatment with immune checkpoint inhibitors.

## Conclusions

In this study we first reported the intense preclinical antitumor activity of CSPG4-CAR.CIK against melanoma, confirming that such effect stands even when target tumors display low or defective expression of HLA class I molecules, a condition associated with resistance to checkpoint inhibitors in melanoma patients. Our findings support CSPG4 as a valuable target for CAR CIK-based immunotherapy in melanoma. Overall, the results outlined in this study provide strong translational rationale for the design of clinical trials investigating CSPG4-CAR.CIK within the challenging setting of patients with metastatic melanoma who do not respond or relapse to treatment with immune checkpoint inhibitors.

## Data Availability

Public data sharing is not applicable to this article as no datasets were generated or analyzed during the current study. All data generated in this study are available from the authors upon reasonable request.

## Abbreviations

APM: Antigen Processing Machinery
β2m: β2 Microglobulin
CAR: Chimeric Antigen Receptor
CD: Cluster of differentiation
CIK: Cytokine Induced Killer
CM: Central memory
CSPG4: Chondroitin Sulfate Proteoglycan 4
CXCR3: C-X-C motif chemokine receptor 3
E/T: Effector to target
EM: Effector memory
Erp57: Endoplasmic reticulum resident protein 57
HLA-I: Human Leukocyte Antigen class I
ICI: Immune checkpoint inhibitors
IFN: Interferon
LMP2/7/10: Low molecular weight protein 2/7/10
MEK: Mitogen-activated protein kinase kinase
MFI: Mean fluorescence intensity
MIC A/B: MHC class I chain-related protein A/B
N: Naive
NK: Natural killer
NKG2D: Natural killer group 2 receptor
NSG: NOD *scid* gamma
PBMCs: Peripheral blood mononuclear cells
SNV: Single nucleotide variant
TA: Tumor antigen
TAP1/2: Antigen peptide transporter 1/2
ULBP2-5–6: UL16-binding protein2-5–6
